# Fabrication and Electrocatalytic Activity of Fe-Cu/C Composites Based on Copper Ferrite Modified with Graphene Oxide and Graphitic Carbon Nitride

**DOI:** 10.3390/ma19112273

**Published:** 2026-05-27

**Authors:** Yakha A. Vissurkhanova, Nina M. Ivanova, Yelena A. Soboleva, Zainulla M. Muldakhmetov

**Affiliations:** 1 Institute of Organic Synthesis and Coal Chemistry, Karaganda 100000, Kazakhstan; nmiva@mail.ru (N.M.I.); esoboleva-kz@mail.ru (Y.A.S.); iosu.rk@mail.ru (Z.M.M.); 2 Department of Chemistry, Karaganda National Research University Named After Academician Ye. A. Buketov, Karaganda 100024, Kazakhstan

**Keywords:** copper ferrite, graphene oxide, graphitic carbon nitride, electrochemical reduction, Fe-Cu/C composites–catalysts, electrocatalytic hydrogenation

## Abstract

**Highlights:**

- CuFe_2_O_4_/Carbon materials composites were synthesized via co-precipitation and heat treatment.
- The rGO, g-C_3_N_4_, and rGO + g-C_3_N_4_ carbon materials were used for composite preparation.
- The phase composition of heat-treated CuFe_2_O_4_ is influenced by carbon material type.
- Fe-Cu/C composites form after electrochemical reduction of CuFe_2_O_4_/CM composites.
- Fe-Cu/C composites show high catalytic activity in acetophenone electrohydrogenation.

**Abstract:**

A facile co-precipitation method was employed to synthesize copper(II) ferrite composites with carbon materials (reduced graphene oxide, graphitic carbon nitride, and their mixture), followed by heat treatment at 700 °C. To obtain Fe-Cu-containing catalysts, copper ferrite composites were electrochemically reduced. Structures, compositions, and morphologies of the composites were studied using scanning electron microscopy, X-ray diffraction techniques, and thermogravimetric analysis. The results showed that graphitic carbon nitride had the strongest effect on the phase composition of copper ferrite. Crystalline phases of reduced copper and iron metals appear in the CuFe_2_O_4_/g-C_3_N_4_ composite during the annealing process, facilitating further complete electrochemical reduction of copper ferrite and shortening its duration. The resulting Fe-Cu/C composites were used as catalysts in the electrohydrogenation of acetophenone as a model compound. The activation of the cathode with Fe-Cu/C catalysts increases the rate of acetophenone hydrogenation and leads to the selective formation of a single product, 1-phenylethanol, in high yields.

## 1. Introduction

Replacing expensive catalysts containing noble metals (platinum, gold, etc.) and developing catalytic systems based on readily available transition metals for use in the chemical industry remain major challenges in heterogeneous catalysis and electrocatalysis. Transition metals, such as iron (Fe) and copper (Cu), represent an attractive alternative due to their abundance in the Earth’s crust, low cost, and environmental safety. Much attention has been paid to the development of iron- and copper-containing catalytic systems [1,2]. Of particular interest are bimetallic Fe-Cu systems, in which the presence of copper enhances the activity and improves the selectivity of such composites due to the electron interaction between the two metals [3,4,5]. Furthermore, due to their magnetic properties, Fe-Cu composites can be easily separated from the reaction medium using an external magnet, ensuring their complete recovery and repeated use.

To prepare Fe-Cu catalytic systems, various physical, chemical, mechanochemical, thermal, and electrochemical methods are used [5,6,7]. Metal powders, metal salts, metal oxides, and organometallic compounds are usually used as precursors for obtaining iron-copper composites [5,8].

Often, during the preparation of Fe-Cu-containing nanocomposites, metal oxidation or particle aggregation during synthesis occurs. Therefore, Fe-Cu nanoparticles are supported on organic and inorganic supports or various stabilizers are used. Among carbon-containing supports, graphene and its derivatives occupy a special place due to their stability, high electrical conductivity, and a large specific surface area [9,10,11,12,13]. The literature contains examples of the preparation of bimetallic Fe-Cu composites on graphene supports prepared using various methods [14,15,16]. Another frequently used carbon-containing support for catalytically active metal nanoparticles is graphite-like carbon nitride, g-C_3_N_4_. Fe-Cu composites obtained with the participation of g-C_3_N_4_ are used both as catalysts and photocatalysts [17,18,19]. The combination of GO and g-C_3_N_4_ in the synthesis of metal-carbon composites can lead to a synergistic effect: GO provides electrical conductivity, and g-C_3_N_4_ promotes the dispersion of active sites. In addition, from rGO + g-C_3_N_4_ mixtures, the structurally modified graphene with N-doped carbon is obtained, which improves interaction with metal particles and, for example, exhibits high catalytic activity as a metal-free electrocatalyst in hydrogen and oxygen evolution reactions [20].

One method for producing Fe-Cu catalysts is the reduction of copper(II) ferrite, CuFe_2_O_4_, with reducing gases (hydrogen, carbon monoxide, and methane) at a temperature range of 200–1000 °C. The reduction of copper ferrite with hydrogen was studied in [21,22,23,24,25]. It was found that the reduction of CuFe_2_O_4_ occurs in two stages at temperatures of 200 °C and 400 °C. Initially, metallic copper and magnetite are formed [24]:

3CuFe_2_O_4_ + 4H_2_ → 3Cu^0^ + 2Fe_3_O_4_ + 4H_2_O. (1)

Subsequently, at 400 °C, a mixture of copper and wustite, FeO, is first formed, from which iron, α-Fe, is then reduced [24,25]. The bimetallic Fe-Cu composites prepared in this way, which have magnetic properties, are used as catalysts in various chemical processes.

It should be noted that copper ferrite and its composites with rGO and g-C_3_N_4_ are also in demand as effective catalytic systems. For instance, CuFe_2_O_4_/rGO composites have shown multifunctionality as high-performance electrodes for flexible supercapacitors and catalysts for nitroaromatic reduction [26]. CuFe_2_O_4_/rGO composites showed high catalytic and photocatalytic activity in the degradation of phenol, ammonium perchlorate, and other pollutants [27,28,29,30,31]. CuFe_2_O_4_/g-C_3_N_4_ composites were studied as photocatalysts for producing H_2_ by splitting water under the influence of visible light, as well as in the degradation of drugs and other pollutants [32,33,34,35,36]. However, there is no data in the literature on the creation of Fe-Cu/C catalysts based on them.

In our previous papers [37,38], Fe-Cu composites were produced from copper CuFe_2_O_4_ and copper-zinc Cu_0.5_Zn_0.5_Fe_2_O_4_ ferrites by their reduction in an electrochemical system in an aqueous-alkaline catholyte at a temperature of 30 °C. It was found that the presence of polymeric particle stabilizers (polyvinyl alcohol, polyvinylpyrrolidone) in the reaction medium for the co-precipitation of precursors (oxides and hydroxides of iron and copper) affects the phase composition of the metal ferrites formed during heat treatment. This study focuses on the fabrication of iron-copper composites based on copper ferrite modified with graphene oxide, graphitic carbon nitride and their mixture. The choice of these carbon materials as modifiers is justified by their ability to act as structural templates that effectively inhibit the aggregation of metal oxides and hydroxides during copper and iron cations co-precipitation and subsequent thermal treatment. The purpose of this research is to study the specific influence of carbon modifiers on the structural and phase transformations of copper ferrite during heat treatment, their electrochemical reduction with the formation of Fe-Cu/C and Fe-Cu/N-C composites, and their electrocatalytic activity in the electrohydrogenation of organic compounds using acetophenone as an example.

## 2. Materials and Methods

### 2.1. Materials

All chemicals were of analytical grade and used as received without further purification. Graphite (carbon content ~99%, particle sizes of 0–50 μm) was purchased from “Temsen” LLP (Lysva, Perm Krai, Russia). Sulfuric acid (H_2_SO_4_, 92% solution), potassium permanganate (KMnO_4_, 99%), hydrochloric acid (HCl, 37% solution), and hydrogen peroxide (H_2_O_2_, 30% solution) were provided by “Karagandareactivsbyt” LLP (Karaganda, Kazakhstan). Dicyandiamide was purchased from Sigma-Aldrich (Saint Louis, MO, USA). Copper(II) nitrate trihydrate (Cu(NO_3_)_2_·3H_2_O), iron(III) nitrate nonahydrate (Fe(NO_3_)_3_·9H_2_O) and sodium hydroxide (NaOH, 99%) were supplied by “Ridder” LLP (Karaganda, Kazakhstan). Distilled water was used for the preparation of all aqueous solutions.

### 2.2. Preparation of Carbon Materials

#### 2.2.1. Preparation of GO

GO was prepared via a modified Hummers method [39]. Briefly, 8 g of graphite was dispersed in 200 mL of H_2_SO_4_ under constant stirring for 4 h at room temperature. Subsequently, 24 g of KMnO_4_ was slowly added to the mixture in an ice-water bath and stirred for 8 h. Then, a mixture of 40 mL H_2_O_2_ and 400 mL distilled water was added dropwise under constant stirring for 1 h. The resulting product was filtered and washed with 400 mL of 5% HCl solution and 2000 mL of distilled water. As-synthesized loose black product dried at 50 °C.

#### 2.2.2. Preparation of g-C_3_N_4_

The bulk of g-C_3_N_4_ was prepared by direct heating of dicyandiamide according to the reported literature [40]. Typically, 12 g of dicyandiamide (DCDA) was put into an alumina crucible with a cover and heated at a rate of ~10 °C min^−1^ and kept at 550 °C for 3 h under air. The obtained yellow-coloured material was ground to get a fine powder.

#### 2.2.3. Preparation of rGO + g-C_3_N_4_ Mixture

The rGO + g-C_3_N_4_ mixture was prepared via a calcination method using a mixture of dicyandiamide and graphene oxide. Briefly, 6 g dicyandiamide and 6 g graphene oxide were mixed and placed into an alumina crucible with a cover. After annealing at 550 °C for 3 h in air, the resulting powder was grounded.

### 2.3. Synthesis of CuFe_2_O_4_ and CuFe_2_O_4_/Carbon Material Composites

Firstly, 7.2 g of carbon materials (CM) powder was dispersed in 100 mL of distilled water and stirred for 1 h at ambient temperature. The amount of carbon materials was chosen to match the theoretical yield of copper ferrite (7.2 g) to obtain a 1:1 mass ratio in the resulting composite. Separately, 0.03 mol of Cu(NO_3_)_2_·3H_2_O and 0.06 mol of Fe(NO_3_)_3_·9H_2_O were dissolved in 200 mL of distilled water. The above two mixtures were put together and stirred for 1 h. Subsequently, 2M NaOH solution was gradually added up to pH 12. After stirring for 1 h, the reaction mixture was filtered and washed with hot distilled water. The resulting sample was thermally treated at 700 °C for 2 h with limited oxygen access (in covered crucibles). Finally, the product was milled using a Tube Mill control electric mill. Pure copper ferrite was synthesized following the same procedure without the addition of carbonaceous materials.

### 2.4. Electrochemical Experiments

The synthesized CuFe_2_O_4_ and CuFe_2_O_4_/CM composites were studied in two sequential stages. In the first stage, the composites were electrochemically reduced in a diaphragm cell with an MK-40 cation-exchange membrane in a 2% NaOH solution at a current of 2.5 A and a temperature of 30 °C. The powder composite (1 g) was deposited onto a horizontally positioned copper cathode (0.09 dm^2^), which served as a conductive substrate and was held by an external magnet (B = 0.05 T); a platinum mesh served as the anode. In the second stage, acetophenone (APh, 0.162 mol/L) was introduced into the cathode compartment, and electrocatalytic hydrogenation was performed in an alcohol-aqueous-alkaline catholyte under the same conditions. The working electrode potential was not monitored; however, the overall cell voltage was recorded during the experiments. It was found that the voltage decreased over time during the processes: from approximately 40 to 17 V during the reduction of metal cations, and from 20 to 16 V during the electrocatalytic hydrogenation of acetophenone. The observed voltage decrease is due to a decrease in system resistance, changes in the composition of the reaction medium, and changes in the kinetics of electrode processes. The volumes of evolved gases (H_2_ and O_2_) were monitored to evaluate the completeness of metal cation reduction in the first stage, and in the second stage, the average hydrogenation rate (W, mL H_2_/min) for period of α = 25%, the hydrogen utilization factor (η, %), and the degree of APh conversion (α, %) to the main product 1-phenylethanol (1-PhEt) were calculated. After the completion of the experiment, the hydrogenation products were extracted from the catholytes and analyzed by gas chromatography.

### 2.5. Characterization

The structural properties of the synthesized composites were investigated by X-ray diffraction (XRD) analysis using a Rigaku SmartLab diffractometer (Rigaku, Tokyo, Japan) with CuK_α_ radiation in the 2θ range of 5–90°. Morphological features of these composites were studied using a scanning electron microscope (SEM) JSM-IT 800 (JEOL, Tokyo, Japan). The samples were analyzed after deposition of a conductive gold layer. The elemental microanalysis of the ferrite samples was performed using the Ultim Extrime energy-dispersive detector (Oxford Instruments, Abingdon, UK). Thermal gravimetric analysis (TGA) was carried out using a LabSYS evo TGA/LTA/DSC analyzer (Setaram Instrumentation, Caluire-et-Cuire, France) at a heating rate of 10 °C/min in the temperature range of 25–900 °C in air. Chromatographic analyses of the catholyte extracts (with chloroform as an extractant) after the completion of electrocatalytic hydrogenation of APh were performed on a Crystallux-4000 chromatograph (NPF Meta Chrom, Yoshkar-Ola, Russia).

## 3. Results and Discussion

**Figure 1:**
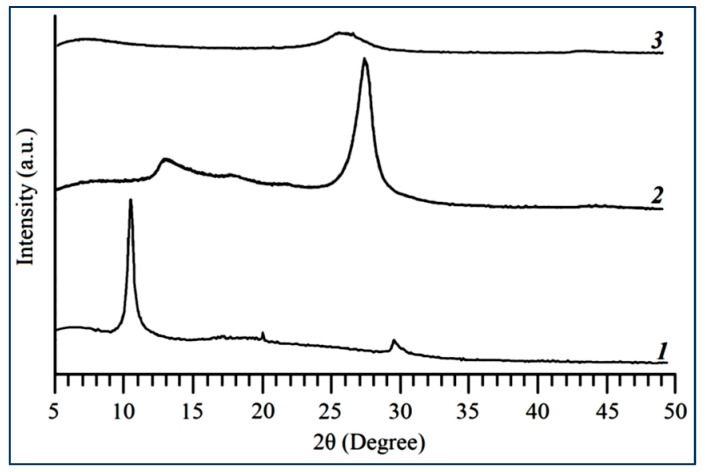
XRD patterns of carbon materials as components in CuFe_2_O_4_/carbon materials composites: *1*—GO, *2*—g-C_3_N_4_, and *3*—rGO + g-C_3_N_4_ mixture.

The XRD patterns of the as-prepared carbonaceous materials used for CuFe_2_O_4_/CM composite synthesis are shown in Figure 1. The XRD pattern of GO (Figure 1, curve *1*) is characterized by a prominent (001) reflection at 2θ ≈ 11.3°, confirming increased interlayer spacing due to oxygen functionalization.

The content of oxygen-containing groups in graphene oxide synthesized by the modified Hummers method was determined as previously described in our work [39], following the procedures detailed in [41,42]. The Raman spectrum of GO, along with its description, is provided in the Figure S1 of the Supplementary Materials [26,43]. For the graphitic carbon nitride prepared by the thermal treatment of DCDA at 550 °C, the peaks at ~13.25° and ~27.42° (2θ) are assigned to the (100) and (002) planes, respectively (Figure 1, curve *2*). The angle values are in good agreement with the literature data for g-C_3_N_4_ [44]. The rGO + g-C_3_N_4_ mixture was prepared from dry powders of GO and DCDA in a 1:1 ratio and heated at 550 °C. The low-intensity peak in the XRD pattern for the mixture (Figure 1, curve *3*) combines two closely related phases: one for reduced graphene oxide with an angle of 2θ = 25.6°, and the other for g-C_3_N_4_ with an angle of 2θ = 26.7°. The observed broadening and low intensity indicate a high degree of structural disorder and mutual exfoliation of the carbon layers during the simultaneous reduction of GO and polycondensation of DCDA. This suggests the formation of a disordered heterostructure with a high surface area rather than a new crystalline phase, providing a robust matrix for the subsequent growth of CuFe_2_O_4_ nanoparticles [45].

After co-precipitation, the precursors of copper ferrite (copper(II) and iron(III) hydroxides) and their composites with carbon materials were heat-treated at 700 °C in air. This heat treatment (HT) temperature was selected based on our previous studies [37], which demonstrated that copper ferrite samples synthesized in the presence of polymer stabilizers and treated at 700 °C exhibit optimal performance during subsequent thermal and electrochemical reduction.

**Figure 2:**
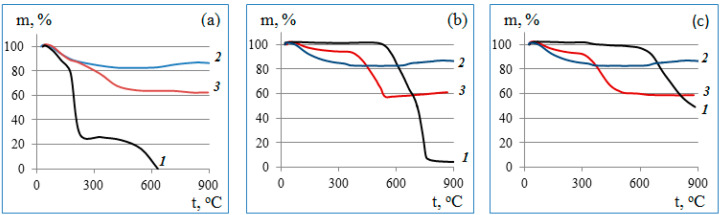
TG curves for (**a**) GO (*1*), CuFe_2_O_4_ (*2*) and CuFe_2_O_4_/rGO (*3*) precursors; (**b**) g-C_3_N_4_ (*1*), CuFe_2_O_4_ (*2*) and CuFe_2_O_4_/g-C_3_N_4_ (*3*) precursors; (**c**) rGO + g-C_3_N_4_ (*1*), CuFe_2_O_4_ (*2*) and CuFe_2_O_4_/rGO + g-C_3_N_4_ (*3*) precursors.

Thermogravimetric analyses (TGA) in air were carried out for the composite precursors obtained after the co-precipitation procedure without and in the presence of carbon materials, as well as for the carbon components added to the reaction medium (Figure 2).

According to the TGA data, the main thermal decomposition of graphene oxide GO (Figure 2a, curve *1*) occurs in a narrow temperature range of ~180–203 °C with a sharp mass loss (up to 60% of the initial mass), apparently under the influence of decomposing oxygen-containing groups. Then, as the temperature increases to ~490 °C, stable behaviour of the already reduced graphene oxide is observed with a mass loss of only 10%. In the temperature range of ~500–630 °C, the final combustion of carbon residues interacting with atmospheric oxygen occurs. The curve of copper ferrite precursors (Figure 2a, curve *2*) shows one significant decline, starting at 50 °C and continuing to 360 °C with a mass loss of ~17%, caused by the evaporation of surface water and water from the decomposing metal hydroxides, as well as the final formation of the copper ferrite structure. Above 640 °C, the copper ferrite exhibits thermal stability, and in the range of 650–900 °C, its mass somewhat increases. The CuFe_2_O_4_/rGO composite precursors exhibit a different behaviour (Figure 2a, curve *3*). Initially, water evaporates at temperatures up to 200 °C, and oxygen-containing groups in GO decompose. The removal of oxygen-containing groups occurs partially (weight loss in this temperature range is ~13%), apparently due to interaction with metal oxides (CuO, Fe_2_O_3_) formed during the decomposition of these metal hydroxides. Copper ferrite then forms at temperatures up to 450 °C, with a further weight loss of ~20%. Beyond this temperature, the CuFe_2_O_4_/rGO composite exhibits thermal stability up to 900 °C.

Figure 2b shows the TGA curves for the thermal formation of the CuFe_2_O_4_/g-C_3_N_4_ composite and copper ferrite from their precursors, as well as for g-C_3_N_4_ prepared at 550 °C. Carbon nitride (Figure 2b, curve *1*) loses practically no weight at temperatures below 540 °C, indicating its high thermal stability. However, a further increase in temperature up to 760 °C leads to a significant mass loss, leaving ~7% residual apparently N-doped carbon. Compared to g-C_3_N_4_, the thermal decomposition of the CuFe_2_O_4_/g-C_3_N_4_ composite occurs at lower temperatures (Figure 2b, curve *3*). First, in the region of 100 °C, the surface liquid leaves, and then water is released from the metal hydroxides. The mass of the sample decreases smoothly by only ~6% up to a temperature of 360 °C. The main significant decrease in the composite mass (~34–35%) occurs in the temperature range of 360–550 °C. Since the formation of pure copper ferrite is complete at 350 °C, the main decrease in the TGA curve of the composite is caused by the earlier decomposition of g-C_3_N_4_, due to the influence of released water, as well as oxygen released from the copper ferrite during metal reduction, which, in turn, is caused by the decomposition products of carbon nitride. The residual mass of the CuFe_2_O_4_/g-C_3_N_4_ composite at 700 °C was ~63–64% of the initial mass of its precursors.

The carbonaceous support for the CuFe_2_O_4_/(rGO + g-C_3_N_4_) composite was produced by thermal treatment of a mixture of GO and DCDA at 550 °C. Therefore, according to the TGA data (Figure 2c, curve *1*), this mixture is thermally stable up to 535 °C. Then, it began to decompose up to 900 °C, but the mass loss was not as significant as in the case of GO and g-C_3_N_4_ separately, and amounted to ~47%. Obviously, the decrease in the mass of the CuFe_2_O_4_/(rGO + g-C_3_N_4_) composite precursors with an increase in temperature to 430 °C (Figure 2c, curve *3*) is due to changes in the copper ferrite precursors interacting with the carbon base. Consequently, the thermal stability of this composite established in the region of 507 °C was less than that of copper ferrite itself, and the mass loss at 700 °C was ~42%.

**Figure 3:**
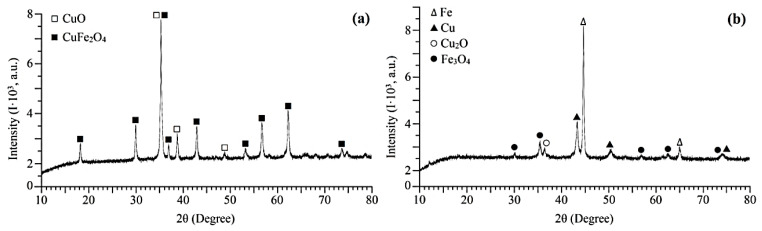
XRD patterns of CuFe_2_O_4_ (**a**) after HT at 700 °C and (**b**) after APh electrocatalytic hydrogenation.

**Figure 4:**
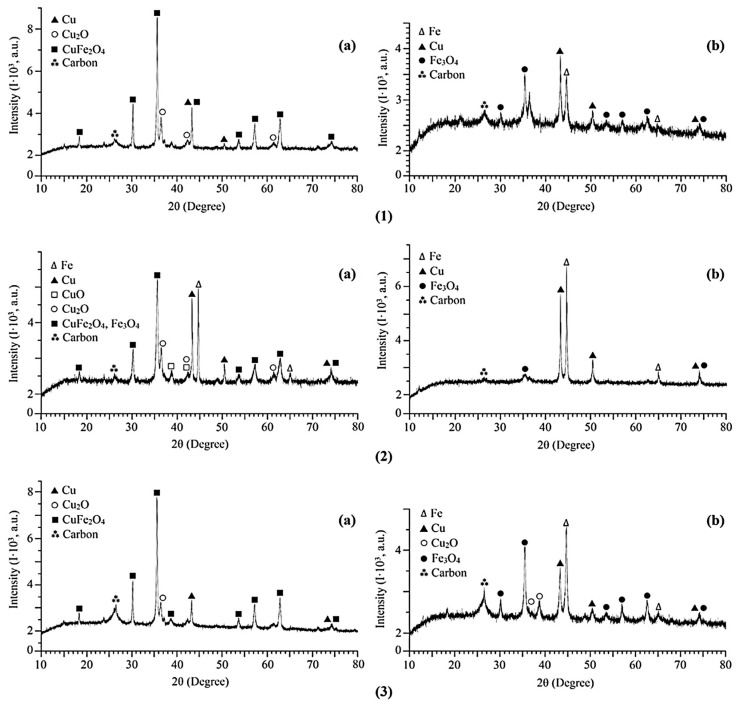
XRD patterns of CuFe_2_O_4_/rGO (1), CuFe_2_O_4_/g-C_3_N_4_ (2) and CuFe_2_O_4_/(rGO + g-C_3_N_4_) (3) composites (**a**) after HT at 700 °C and (**b**) after electrocatalytic hydrogenation of APh.

Notably, the TGA results for all CuFe_2_O_4_/rGO, CuFe_2_O_4_/g-C_3_N_4_, and CuFe_2_O_4_/(rGO + g-C_3_N_4_) composites show approximately the same residual mass (~59–64%) in the 700 °C region. However, the compositions of these composites after HT at 700 °C in a muffle furnace differed, which was established by X-ray diffraction analyses (Figure 3 and Figure 4).

In the XRD pattern of pure CuFe_2_O_4_ (700°C) (Figure 3a), peaks at 2θ = 18.2°, 30.0°, 35.3°, 36.9°, 42.8°, 56.6°, and 62.2° correspond to the planes (111), (220), (311), (202), (400), (511), and (440) of the cubic crystal of CuFe_2_O_4_. Additionally, the characteristic peaks located at 2θ = 32.5°, 35.5°, 38.8°, and 48.7° are assigned to CuO. After the electrochemical reduction of copper ferrite and the subsequent use of the resulting composite in the electrocatalytic hydrogenation of acetophenone, the crystalline phases of iron (Fe^0^) and copper (Cu^0^) (in smaller amounts), as well as their oxides Fe_3_O_4_ and Cu_2_O in minor quantities, were detected (Figure 3b). That is, in the electrochemical system, copper ferrite is reduced and a composite with a predominant content of iron and copper is formed, exhibiting magnetic properties, which can be used as a catalyst.

The CuFe_2_O_4_/rGO phase composition after HT (Figure 4(a1)) includes the crystalline phases of copper ferrite itself, reduced graphene oxide formed during the heat treatment, accompanied by partial mass loss, as well as small amounts of copper(I) oxide and metallic copper likely formed via the partial reduction of CuO by rGO decomposition products. In the CuFe_2_O_4_/rGO composite after electrochemical experiments (Figure 4(b1)), the content of reduced copper increased, partially reduced iron appeared, and oxide phases (Cu_2_O and CuFe_2_O_4_ or Fe_3_O_4_) were preserved. Thus, in this composite, the electrochemical reduction of the metal cations proceeds incompletely and to a lesser extent than in pure copper ferrite (Figure 3b). To distinguish between the crystalline phases with identical diffraction angles (CuFe_2_O_4_ and Fe_3_O_4_), Rietveld analysis was performed on the X-ray diffraction patterns of all synthesized CuFe_2_O_4_/C samples and Fe-Cu/C composites (Figures S2–S5 in the Supplementary Materials).

**Figure 5:**
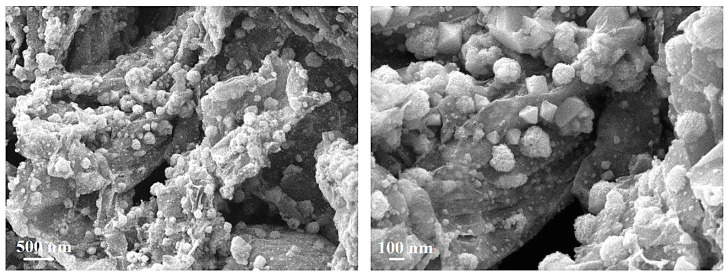
SEM images of the CuFe_2_O_4_/rGO (700 °C) composite.

In the SEM images of CuFe_2_O_4_/rGO composite after HT (Figure 5), the crystallites of CuFe_2_O_4_ are clearly visible in the form of tetrahedral pyramids of various sizes (40–120 nm), as well as larger, loose agglomerates composed of copper(I) oxide platelets attached to multilayer graphene sheets. The element distribution maps for one of the areas of the CuFe_2_O_4_/rGO (700 °C) composite (Figure S6 in the Supplementary Materials) show that Fe and Cu particles are uniformly dispersed throughout the carbonaceous matrix.

However, some local agglomerates consisting of Fe and Cu are also observed, where the elemental mapping reveals specific areas with high signal density for both Cu and O. The strong spatial correlation between these elements suggests the presence of copper (I) oxide species in these regions.

The CuFe_2_O_4_/g-C_3_N_4_ composite synthesized by the co-precipitation method in the presence of graphitic carbon nitride underwent significant structural changes during the subsequent HT (Figure 4(a2)). Its composition still contains crystalline phases of CuFe_2_O_4_ (or Fe_3_O_4_, as their diffraction reflections overlap) and exhibits the appearance of crystalline phases of reduced copper and iron metals with high-intensity peaks, as well as copper oxides. In the electrochemical cell, this composite is then almost completely reduced (Figure 4(b2)). The copper content in the resulting Fe-Cu/N-rGO composite is noticeably higher than that in the bimetallic composite obtained after the electrochemical reduction of the “pure” CuFe_2_O_4_ (Figure 3b).

**Figure 6:**
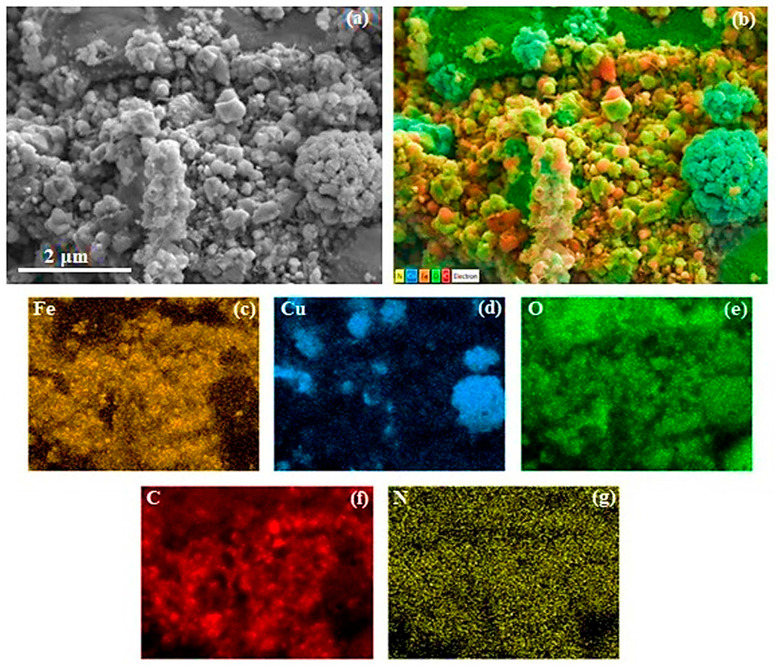
(**a**) SEM image of the CuFe_2_O_4_/g-C_3_N_4_ (700 °C) composite after HT, and (**b**–**g**) elements distribution maps for one of the areas of the composite.

In the SEM image of the CuFe_2_O_4_/g-C_3_N_4_ composite after synthesis and heat treatment (Figure 6a), rather large particles of various shapes are visible, both as individual entities and as agglomerates. The distribution of all elements over this area is fairly uniform, indicating a good interaction between copper ferrite and carbon nitride. However, there are also large spherical agglomerated particles that apparently consist of copper oxides, as shown by the distributions of Cu and O on the elemental map according to the EDS analyses (Figure 6b–g). Additionally, plate-like formations containing elements such as O, N, C, and Na are present. The localized segregation of Fe and Cu, as seen in the EDS maps, correlates well with the XRD data, confirming the presence of secondary phases (CuO, metallic Cu, and Fe) alongside the CuFe_2_O_4_ spinel and N-carbon base.

**Figure 7:**
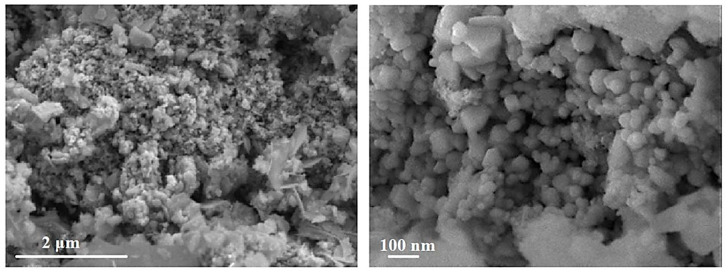
SEM images of the Fe-Cu/N-C composite after electrohydrogenation of APh.

The Fe-Cu/N-C composite formed from CuFe_2_O_4_/g-C_3_N_4_ (700 °C) composite after the electrochemical experiments (Figure 7) contained small, rounded bimetallic particles (~30–80 nm) and plate-like structures that apparently consisted of N-containing carbon base with inclusions of particles of both metals. Many particles exhibit a porous and, in some areas, a mesh-like structure with regions with higher concentrations of copper or iron (Figure S7 in the Supplementary Materials). EDS mapping shows that the round particles containing two metals are covered with a layer of iron, which is due to the high content in the copper ferrite structure and the longer recovery of iron cations. Therefore, the active centers in the studied Fe-Cu catalytic systems are, first of all, iron particles, as well as copper particles in their individual aggregated clusters.

The CuFe_2_O_4_ composite with a mixed carbon matrix of rGO + g-C_3_N_4_ thermally treated at 700 °C (Figure 4(a3)), resulting in the formation of N-rGO, has an almost identical phase composition to that of the CuFe_2_O_4_/rGO composite after HT (Figure 4(a1)). It contains crystalline phases of CuFe_2_O_4_, Cu_2_O, partially reduced copper, and N-doped rGO. For the main N-rGO (002) phase, the diffraction peak is located at 2θ = 26.5°, which is close to that of graphene (~26–27°). Notably, after HT at 700 °C, more carbon material remained in this composite (~40%) than in CuFe_2_O_4_/rGO (~26%) and CuFe_2_O_4_/g-C_3_N_4_ (~16%) composites, which was determined by XRD analysis.

**Figure 8:**
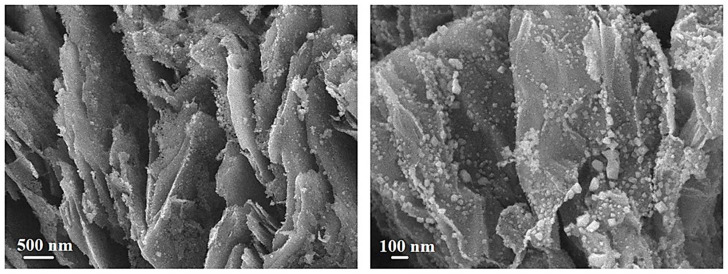
SEM images of the CuFe_2_O_4_/(rGO + g-C_3_N_4_) (700 °C) composite after HT.

A representation of the morphological structure of this CuFe_2_O_4_/(rGO + g-C_3_N_4_) (700 °C) composite is given by its SEM images (Figure 8), which show layers of rGO or N-rGO with adjacent particles of copper ferrite and its oxides. The rGO + g-C_3_N_4_ mixture serves as a multi-functional support that effectively prevents the aggregation and sintering of copper ferrite particles during thermal treatment of the precursor (composed of iron and copper oxides/hydroxides). This results in a loose, porous composite with reduced particle sizes and an enhanced specific surface area. These observations are further supported by EDS elemental mapping (Figure S8 in the Supplementary Materials), which reveals a uniform distribution of N across the rGO layers. Furthermore, the maps highlight regions with a high concentration of Fe, Cu, and O, corresponding to the formation of agglomerated particles. The high local concentration of these elements within the clusters suggests the coexistence of CuFe_2_O_4_ and Cu_2_O phases anchored on the carbon surface. After electrochemical and electrocatalytic experiments, crystalline phases of reduced iron and copper metals appear in this composite, but copper ferrite and/or magnetite phases remained, indicating their incomplete reduction (Figure 4(b3)).

The CuFe_2_O_4_/carbon materials composites were studied for their ability to undergo electrochemical reduction (ECR), and the resulting Fe-Cu composites were examined for the manifestation of electrocatalytic properties in the APh electrohydrogenation (Table 1):

C_6_H_5_–C(O)–CH_3_ + 2e^−^ + 2H_2_O → C_6_H_5_–CH(OH)–CH_3_ + 2OH^−^. (2)

Table 1 shows the following characteristics for Stage 1, including the duration of the ECR of copper ferrite (t_1_) and the volumes of oxygen (V_O2_) additionally released to the volumes of the two gases in a ratio of 2V_H2_ : V_O2_. The given characteristics of the electrocatalytic hydrogenation of APh (Stage 2) are described above in Section 2.4 (additionally, Table 1 shows the duration values of this stage (t_2_)). Chromatographic results for the extracts from the catholyte after the completion of the electrocatalytic hydrogenation of APh are also presented here. A distinctive feature of Stage 2 is that this process was carried out not immediately after completing Stage 1, but after renewing the electrolytes in both compartments of the cell. The characteristics for the electrochemical reduction of APh on a Cu cathode without the deposition of composites are provided for comparison. As shown in Table 1, the electrochemical reduction of acetophenone is not completed (α = 68.3%), and the main reduction product is the trans-isomer of pinacone (trans-2,3-diphenyl-2,3-butanediol), formed as a result of the cathodic hydrodimerization of APh.

Copper ferrite CuFe_2_O_4_ consists of two oxides, CuO and Fe_2_O_3_, and the electrochemical reduction of metal cations apparently begins with them. As shown previously [37], the stepwise (30, 60, 90 min) electrochemical reduction of copper and iron cations in copper(II) ferrite under similar conditions occurs in parallel. Electroreduction of CuO and Fe_2_O_3_ in an alkaline medium can occur both directly with the formation of zero-valent metals and through the formation of intermediate oxides Cu_2_O [46] and magnetite Fe_3_O_4_ [47,48,49], respectively. XRD analyses have shown that iron oxide Fe_2_O_3_ is practically absent in CuFe_2_O_4_/CM samples after thermal treatment and after electrochemical experiments, and Cu_2_O and Fe_3_O_4_ are present as intermediate compounds. In general, the mechanism of electrode reduction of CuFe_2_O_4_ in an alkaline medium can be described by the following possible processes:

Cathode: 3CuFe_2_O_4_ + 6e^−^ + 4H_2_O → 3Cu^0^ + 2Fe_3_O_4_ + 8OH^−^, (3)

CuO + 2e^−^ + H_2_O → Cu^0^ + 2OH^−^, (4)

Cu_2_O + 2e^−^ + H_2_O → 2Cu^0^ + 2OH^−^, (5)

Fe_2_O_3_ + 6e^−^ + 3H_2_O → 2Fe^0^ + 6OH^−^, (6)

Fe_3_O_4_ + 8e^−^ + 4H_2_O → 3Fe^0^ + 8OH^−^, (7)

2H_2_O + 2e^−^ → H_2_ + 2OH^−^. (8)

Anode: 4OH^−^ → O_2_ + 2H_2_O + 4e^−^. (9)

The reduction of magnetite can also occur stepwise (Fe_2_O_3_ → FeO → Fe^0^), but the formation of FeO was not recorded by X-ray analyses.

According to the data in Table 1, the electrochemical reduction of both metal cations in the “pure” copper ferrite sample (weighing 1.0 g and 0.5 g) was accompanied by the release of the largest volume of oxygen and required a relatively long time. In the composites containing various CMs, the metal cations were reduced more rapidly not only because these composites contained a smaller amount of copper ferrite (approximately 0.5 g in 1 g of the cathode-deposited composites), but also because, during the heat treatment (before introduction into the cell), the metal cations were partially reduced (Figure 4a). Thus, the partial reduction of metals during heat treatment under the influence of CMs contributes to a shorter duration of electrochemical reduction and a higher overall metal content in the resulting Fe-Cu/C composites. This is especially noticeable for the CuFe_2_O_4_/g-C_3_N_4_ sample, the thermal and electrochemical reduction of which leads to the formation of a Fe-Cu/N-C composite with a high content of reduced metals, and the copper content is even higher than in reduced pure copper ferrite (Figure 3 and Figure 4(b2)).

In turn, the amount of reduced metals in Fe-Cu/C and Fe-Cu/N-C composites and the initial amount of carbon material significantly influenced their electrocatalytic activity in the APh hydrogenation (Table 1, Stage 2). As shown by the presented data, the Fe-Cu composite catalysts obtained without CM, as well as Fe-Cu/C and Fe-Cu/N-C composites synthesized with smaller amounts of carbon additives (with a ratio of 1:1), exhibited pronounced electrocatalytic activity, providing higher hydrogenation rates and greater APh conversion compared to their electrochemical reduction on a non-activated copper cathode.

The highest catalytic activity among carbon-containing Fe-Cu composites was exhibited by the composite obtained using graphitic carbon nitride, which apparently interacts more strongly with metal oxides during the co-precipitation process, and the products of its thermal decomposition have a greater impact on the formation of metallic phases than the composites containing rGO. Furthermore, the rate of APh hydrogenation using this composite is slightly higher than that of the Fe-Cu composite (weighing 0.5 g) without modification with carbon materials (Table 1). This is possibly due to the higher content of reduced copper in the Fe-Cu/N-C composite (Figure 4(b2)) than in the Fe-Cu composite (Figure 3b). According to the chromatographic analyses, in the absence of catalysts, the reduction of APh proceeds mainly with the formation of dimeric products (Table 1). However, the use of the synthesized Fe-Cu, Fe-Cu/C, and Fe-Cu/N-C catalysts leads to the selective formation of a single product, 1-phenylethanol (1-PhEt in Table 1), with high yields.

## 4. Conclusions

Copper ferrite composites were synthesized by co-precipitation of metal hydroxides from their salts in the presence of carbon materials (GO, rGO + g-C_3_N_4_, g-C_3_N_4_), followed by heat treatment at 700 °C. The partial decomposition of copper ferrite under the influence of graphitic carbon nitride and its decay products during heat treatment was established. The formation of reduced Fe and Cu metals during the heat treatment decreased the duration of the subsequent reduction of these metal cations from copper ferrite in the electrochemical system. The resulting Fe-Cu/C and Fe-Cu/N-C composites with magnetic properties exhibited high electrocatalytic activity in the electrohydrogenation of acetophenone (as a model compound), except of composites with increased rGO and g-C_3_N_4_ contents. The prepared Fe-Cu composites with the addition of carbon materials, as well as Fe-Cu prepared from pure copper(II) ferrite, can be used as catalysts in both electrocatalytic and classical catalytic processes of organic compound transformations.

## Figures and Tables

**Table 1:** Electrochemical reduction of copper(II) ferrite composites and electrocatalytic hydrogenation of acetophenone in the presence of Fe-Cu composites.

Copper Ferrite Composites	ECR of Copper Ferrite (Stage 1)		Electrocatalytic Hydrogenation of APh (Stage 2)							
			t_2_, min	W, mL H_2_/min	η, %	α, %	Composition of Extracts, %			
	t_1_, min	∆V_O2_, mL					1-PhEt	APh	*trans*- Isomer	*cis*- Isomer
Cu cathode	–	–	70	5.1	29.0	68.3	6.0	16.0	46.2	13.6
CuFe_2_O_4_ (m = 0.5 g)	140	74.8	60	9.2	54.2	100.0	99.7	0.3	–	–
CuFe_2_O_4_ (m = 1 g)	220	158.2	40	12.4	72.5	100.0	99.8	0.2	–	–
CuFe_2_O_4_/rGO (1:1)	90	109.2	70	6.6	39.4	100.0	95.3	4.7	–	–
CuFe_2_O_4_/rGO (1:2)	90	73.8	100	2.3	13.0	55.0	61.6	26.7	6.5	3.6
CuFe_2_O_4_/g-C_3_N_4_ (1:1)	50	92.8	40	11.2	65.0	100.0	99.8	0.2	–	–
CuFe_2_O_4_/g-C_3_N_4_ (1:2)	50	72.4	140	2.8	16.1	65.7	69.8	30.2	–	–
CuFe_2_O_4_/(rGO + g-C_3_N_4_) (1:1)	90	98.4	100	8.1	45.8	97.7	98.8	1.2	–	–

## Data Availability

The original contributions presented in this study are included in the article/supplementary material. Further inquiries can be directed to the corresponding author.

## Supplementary Materials

The following supporting information can be downloaded at https://www.mdpi.com/article/10.3390/ma19112273/s1: Figure S1. Raman spectrum of GO; Figure S2. XRD patterns of CuFe_2_O_4_ (a) after HT at 700 °C and (b) after APh electrocatalytic hydrogenation with Rietveld analysis; Figure S3. XRD patterns of CuFe_2_O_4_/rGO composite (a) after HT at 700 °C and (b) after electrocatalytic hydrogenation of APh with Rietveld analysis; Figure S4. XRD patterns of CuFe_2_O_4_/g-C_3_N_4_ composite (a) after HT at 700 °C and (b) after electrocatalytic hydrogenation of APh with Rietveld analysis; Figure S5. XRD patterns of CuFe_2_O_4_/rGO + g-C_3_N_4_ composite (a) after HT at 700 °C and (b) after electrocatalytic hydrogenation of APh with Rietveld analysis; Figure S6. (a) SEM image of the CuFe_2_O_4_/rGO (700 °C) composite after HT, and (b–f) elements distribution maps for one of the areas of the composite; Figure S7. (a) SEM image of the CuFe_2_O_4_/g-C_3_N_4_ (700 °C) composite after electrocatalytic hydrogenation of APh, and (b–g) elements distribution maps for one of the areas of the composite; Figure S8. (a) SEM image of the CuFe_2_O_4_/(rGO + g-C_3_N_4_) (700 °C) composite after HT, and (b–g) elements distribution maps for one of the areas of the composite.

## Abbreviations

The following abbreviations are used in this manuscript:

CM	Carbon materials
GO	Graphene oxide
rGO	Reduced graphene oxide
XRD	X-ray diffraction
DCDA	Dicyandiamide
TGA	Thermal gravimetric analysis
SEM	Scanning electron microscope
EDS	Energy-dispersive X-ray spectroscopy
HT	Heat treatment
ECR	Electrochemical reduction
APh	Acetophenone
1-PhEt	1-phenylethanol
