# Supplementary material for: Fabrication and Electrocatalytic Activity of Fe-Cu/C Composites Based on Copper Ferrite Modified with Graphene Oxide and Graphitic Carbon Nitride
Source: Materials (Basel). 2026 May 27;19(11):2273. doi: 10.3390/ma19112273 (PMC13258157; doi:10.3390/ma19112273)
Supplement: Supplementary file 1 [file materials-19-02273-s001.zip › materials-4295958-supplementary.pdf]

materials

MDPI

*Supplementary Materials*

# Fabrication and Electrocatalytic Activity of Fe-Cu/C Composites Based on Copper Ferrite Modified with Graphene Oxide and Graphitic Carbon Nitride

**Yakha A. Vissurkhanova [1,2,*], Nina M. Ivanova [1], Yelena A. Soboleva [1] and Zainulla M. Muldakhmetov [1]**

[1] Institute of Organic Synthesis and Coal Chemistry, Karaganda, 100000, Kazakhstan; nmiva@mail.ru (N.M.I.); esoboleva-kz@mail.ru (Ye.A.S.); iosu.rk@mail.ru (Z.M.M.)
[2] Department of Chemistry, Karaganda National Research University Named After Academician Ye. A. Buketov, Karaganda, 100024, Kazakhstan; yakhavisurkhanova@bk.ru (Ya.A.V.)
* Correspondence: yakhavisurkhanova@bk.ru

The Raman spectrum of the graphene oxide GO sample (Figure S1) exhibit peaks at 1340 and 1574 cm⁻¹, which are assigned to the D and G bands, respectively [1]. The $I_D/I_G$ intensity ratio for the GO is 0.8. In addition, the peak at 2694 $cm^{-1}$ corresponds to the 2D band, indicating that the synthesized graphene oxide consists of a few-layer structure with certain defects [2]. A minor second-order peak is also observed at ~2905 $cm^{-1}$, arising from the combination of the D and G bands.

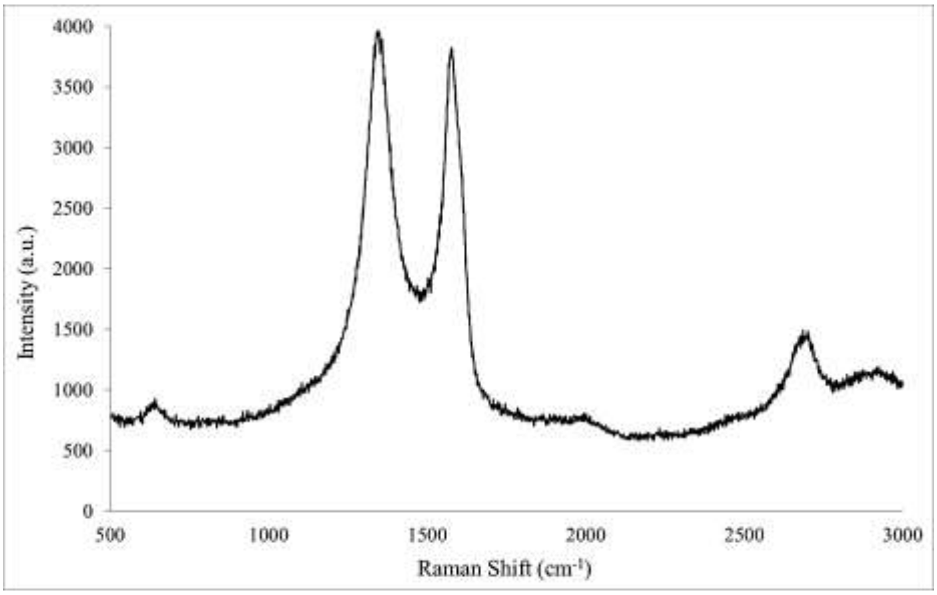


**Figure S1.** Raman spectrum of GO.

 https://doi.org/10.3390/xxxxx

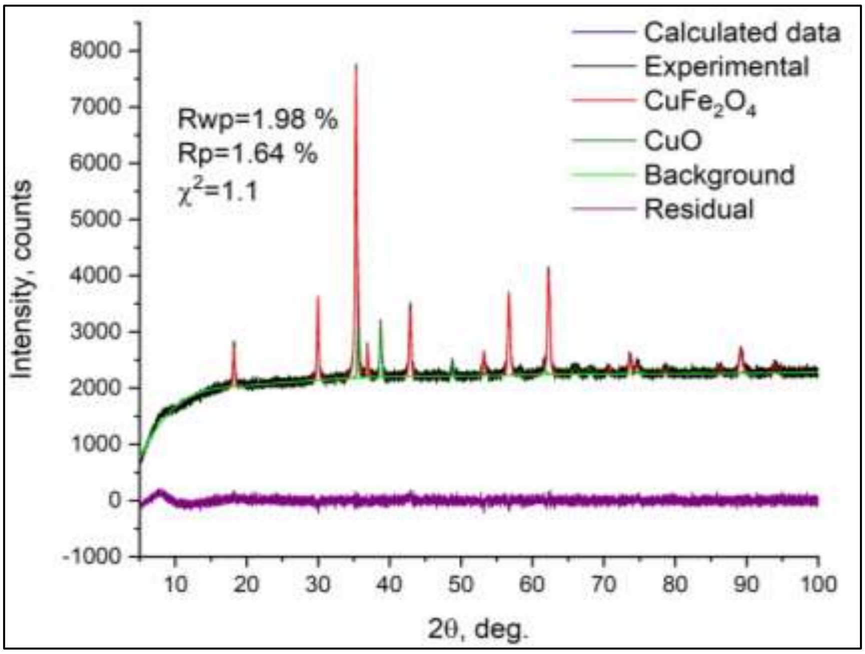


(a)

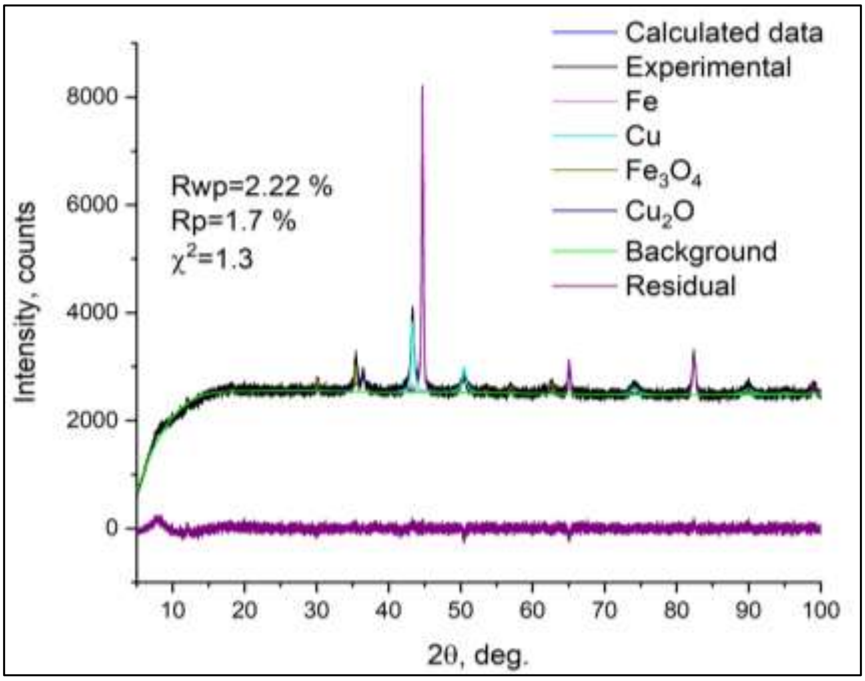


(b)

**Figure S2.** XRD patterns of $CuFe_2O_4$ (a) after HT at 700°C and (b) after APh electrocatalytic hydrogenation **with Rietveld analysis**.

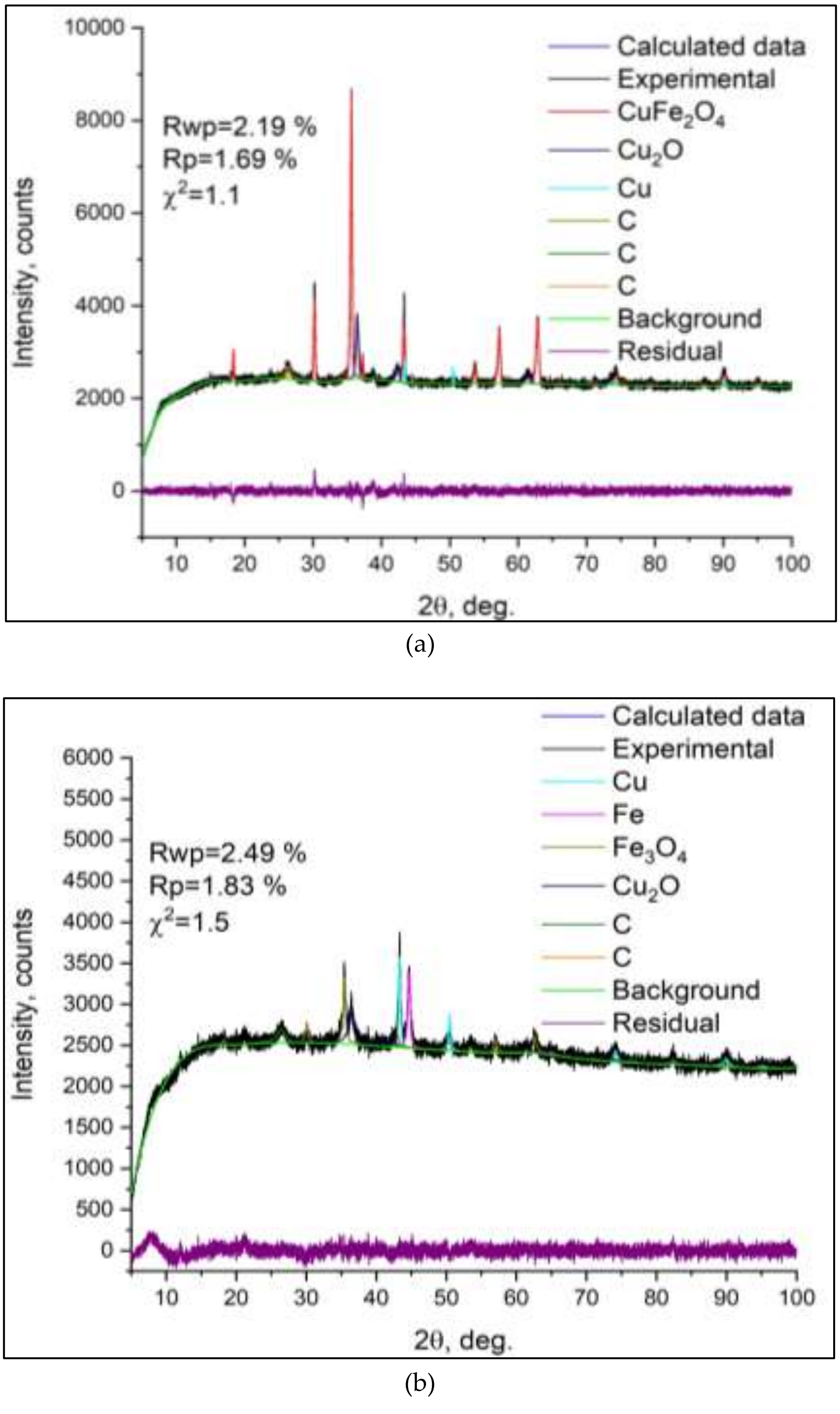


**Figure S3.** XRD patterns of $CuFe_2O_4$/rGO composite (a) after HT at 700°C and (b) after electrocatalytic hydrogenation of APh **with Rietveld analysis**.

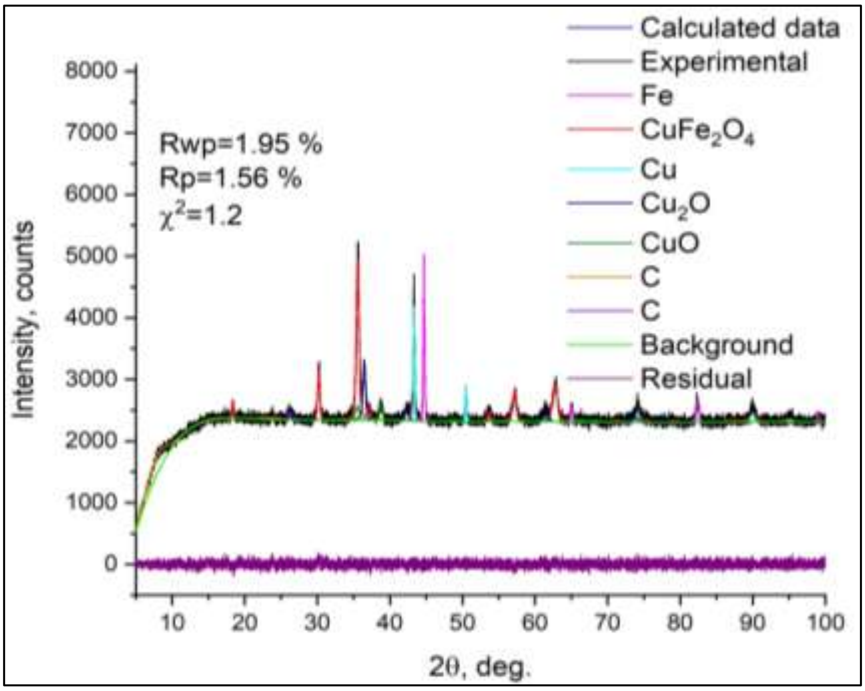


(a)

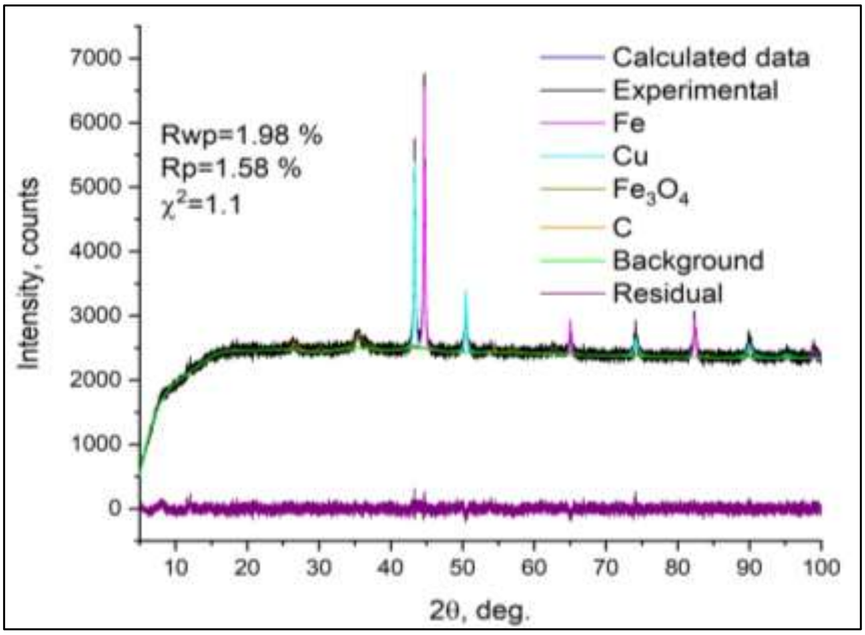


(b)

**Figure S4.** XRD patterns of $CuFe_2O_4$/g-$C_3N_4$ composite (a) after HT at 700°C and (b) after electrocatalytic hydrogenation of APh **with Rietveld analysis**.

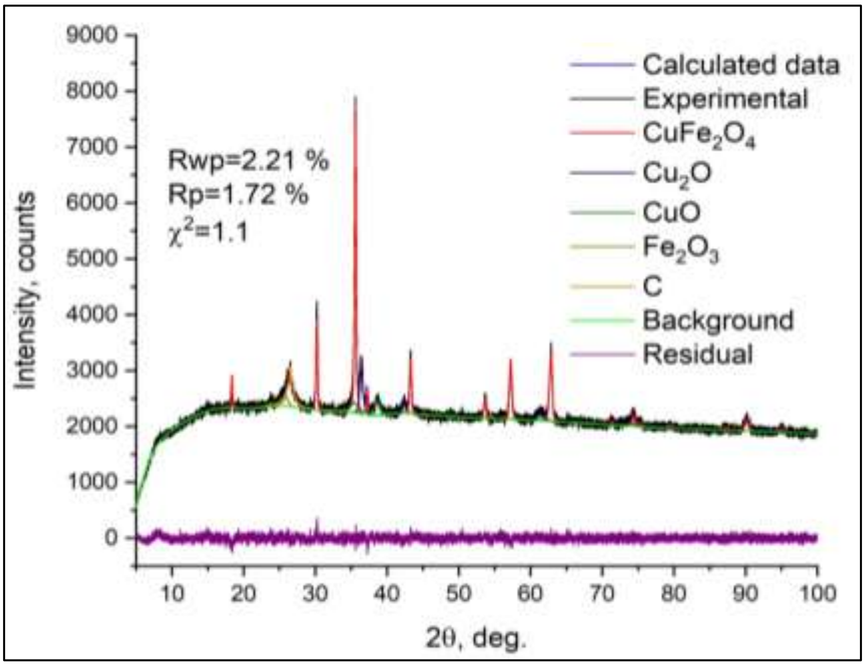


(a)

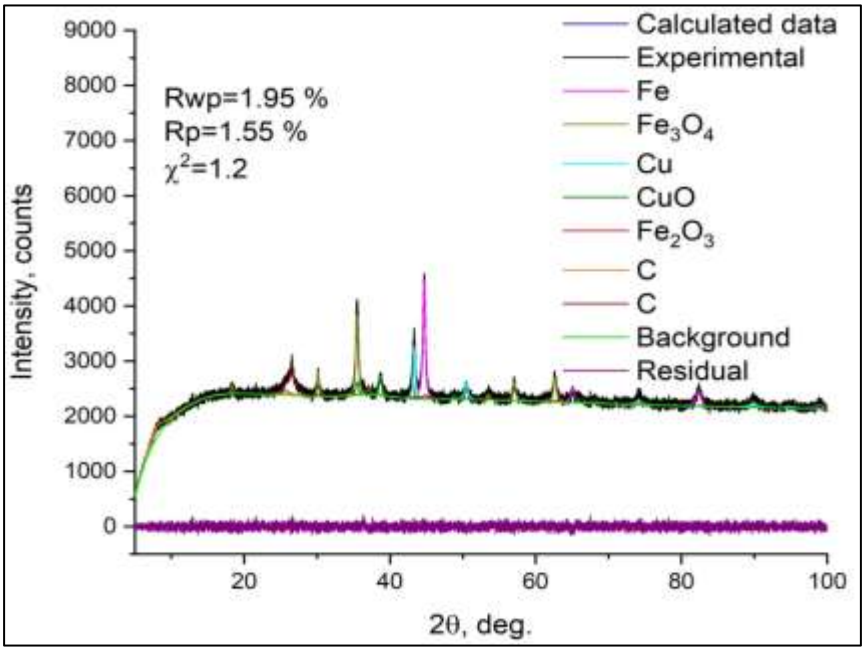


(b)

**Figure S5.** XRD patterns of $CuFe_2O_4$/rGO+g-$C_3N_4$ composite (a) after HT at 700°C and (b) after electrocatalytic hydrogenation of APh **with Rietveld analysis**.

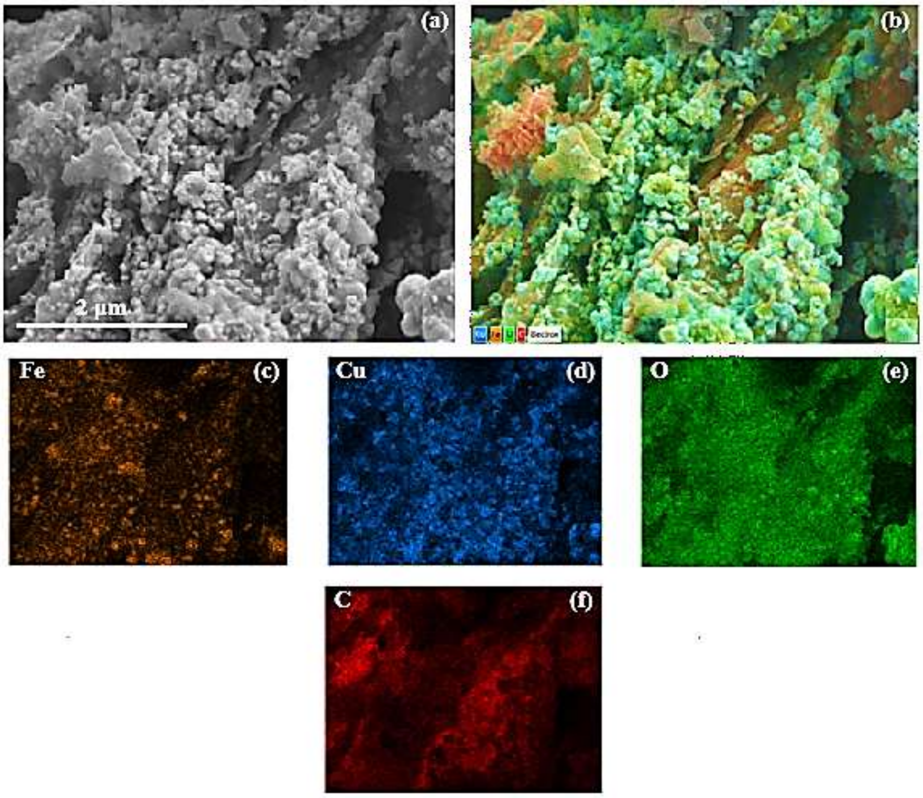


**Figure S6.** (a) SEM image of the $CuFe_2O_4$/rGO(700°C) composite after HT, and (b–f) elements distribution maps for one of the areas of the composite.

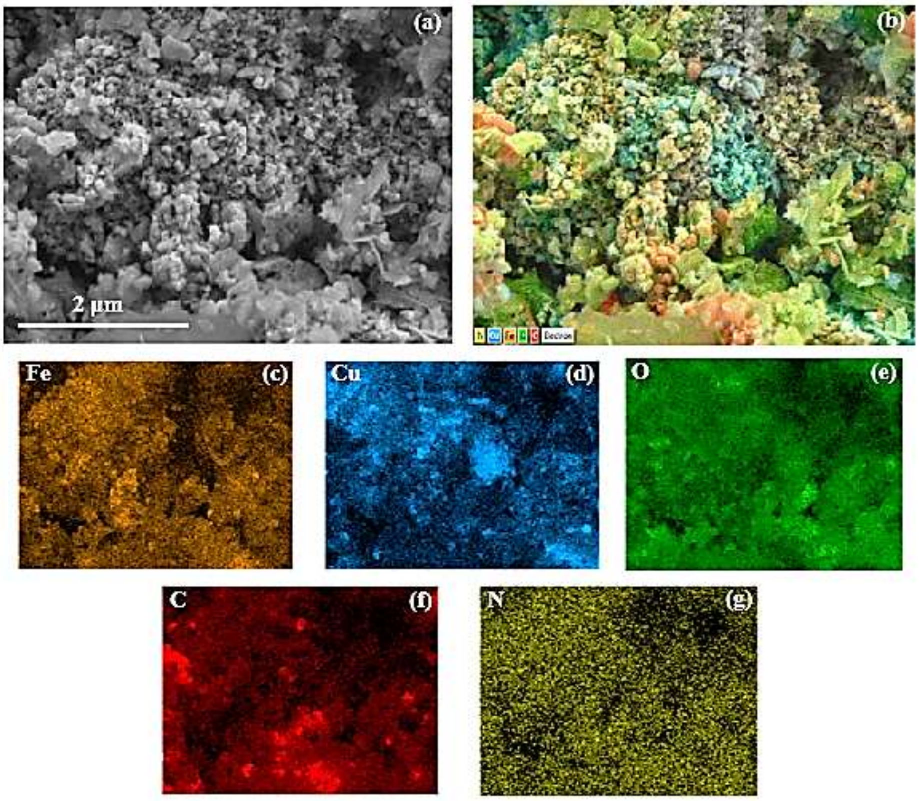


**Figure S7.** (a) SEM image of the the $CuFe_2O_4$/g-$C_3N_4$(700°C) composite after electrocatalytic hydrogenation of APh, and (b–g) elements distribution maps for one of the areas of the composite.

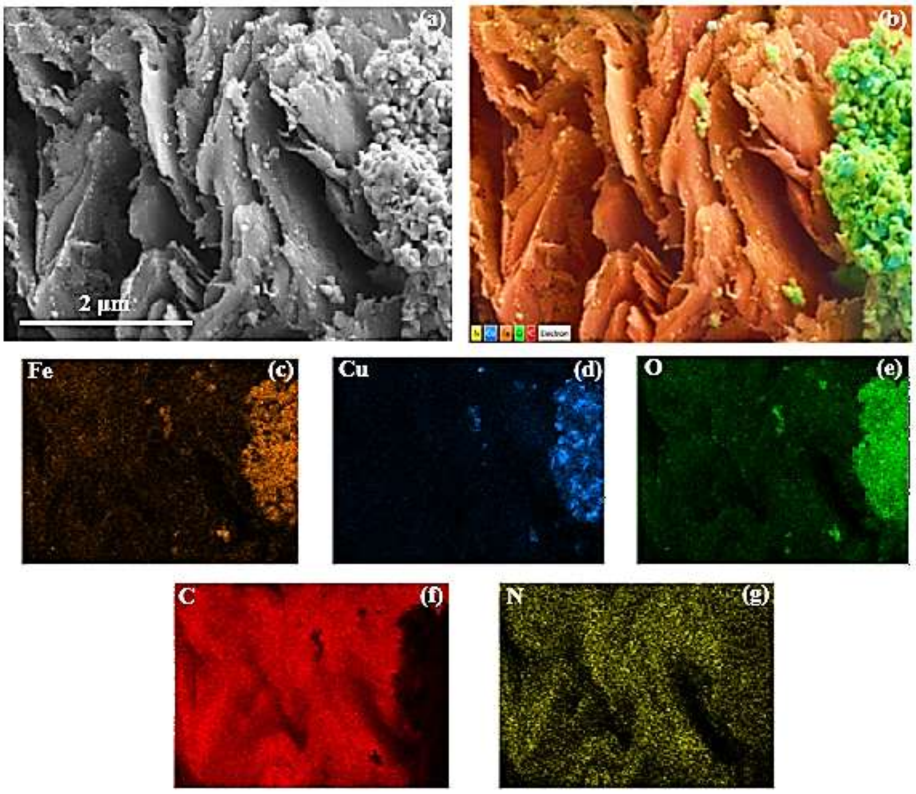


**Figure S8.** (**a**) SEM image of the $CuFe_2O_4$/(rGO + g-$C_3N_4$)(700°C) composite after HT, and (**b**–**g**) elements distribution maps for one of the areas of the composite.

## References

1. Makkar, P.; Gogoi, D.; Roy, D.; Ghosh, N.N. Dual-purpose $CuFe_2O_4$-rGO-based nanocomposite for asymmetric flexible supercapacitors and catalytic reduction of nitroaromatic derivatives. *ACS Omega* **2021**, *6(43)*, 28718–28728. https://doi.org/10.1021/acsomega.1c03377
2. Johra, F.T.; Lee, J.-W.; Jung, W.-G. Facile and safe graphene preparation on solution based platform. *J. Ind. Eng. Chem.* **2014**, 5(20), 2883–2887. https://doi.org/10.1016/j.jiec.2013.11.022
